# Dental spittoon biofilms as reservoirs of antimicrobial resistance: a longitudinal multi-omics study

**DOI:** 10.1128/spectrum.00883-26

**Published:** 2026-09-21

**Authors:** Jesenko Karačić, Lamia Singer, Gabriele Bierbaum, Marijo Parčina, Julius Zilles, Brianne Palmer, Sabina Karačić

**Affiliations:** 1 Institute of Medical Microbiology, Immunology and Parasitology, University Hospital Bonn 39062, Bonn, Germany; 2 Department of Orthodontics, Medical Faculty, University Hospital Bonn 39062, Bonn, Germany; 3 Oral Technology, Medical Faculty, University Hospital Bonn 39062, Bonn, Germany; 4 Department of Paleontology, Bonn Institute of Organismic Biology, University of Bonn 9374, Bonn, Germany; Yan'an University, Yan'an, Shaanxi, China

**Keywords:** dental spittoon biofilms, healthcare-associated biofilms, antimicrobial resistance genes, resistome, carbapenem resistance, plasmid replicons, horizontal gene transfer, multi-omics

## Abstract

Dental chair spittoons are chronically exposed to saliva, aerosols, intermittent water flow, and chemical disinfectants, yet their biofilm ecology and antimicrobial resistance (AMR) dynamics remain poorly defined. We applied longitudinal 16S rRNA gene sequencing, shotgun metagenomics, and culture-based antimicrobial susceptibility testing to biofilms collected across four dental departments at three time points. Community analyses revealed significant temporal succession and department-specific structuring, indicating the establishment of stable, ecologically differentiated biofilm systems. Null-model analysis (Raup-Crick) indicated that community assembly remained predominantly stochastic, although later sampling periods showed modest evidence of increasing ecological filtering. Shotgun metagenomics identified metabolically versatile communities enriched in disinfectant-tolerant environmental taxa, with resistomes dominated by β-lactamases and aminoglycoside-modifying enzymes. Clinically associated plasmid replicons, including IncFII and Col440I, were detected in metagenomically analyzed samples. Culture-based testing of 162 isolates confirmed that 21.7% expressed phenotypic resistance to at least one antimicrobial agent, including multidrug- and carbapenem-resistant representatives of *Pseudomonas* and *Acinetobacter*. Together, these findings position dental spittoons as structured aquatic biofilm ecosystems that maintain viable antimicrobial-resistant populations and clinically relevant plasmid replicons under recurrent disturbance, highlighting their ecological role within the broader built-water resistome.

**IMPORTANCE:**

Biofilms in healthcare environments can act as reservoirs of antimicrobial resistance, yet some potential niches remain poorly studied. Dental chair spittoons are continuously exposed to oral fluids, aerosols, water flow, disinfectants, and residual antimicrobial compounds, creating conditions that may favor biofilm formation and microbial selection. Despite this unique combination of ecological pressures, the microbial communities inhabiting these systems have received little attention. Using a longitudinal multi-omics approach combined with culture-based phenotypic testing, we show that spittoon biofilms harbor diverse microbial communities enriched in antimicrobial resistance determinants, including multidrug-resistant and carbapenem-resistant bacteria. These findings suggest that dental spittoons may represent previously overlooked reservoirs of antimicrobial resistance within clinical environments.

## INTRODUCTION

Biofilms in clinical environments represent persistent microbial reservoirs that contribute to infection persistence and antimicrobial resistance (AMR) dissemination (1, 2). Embedded within a self-produced extracellular matrix, these multispecies communities exhibit increased tolerance to disinfectants, antibiotics, and host defenses, making them difficult to eradicate and central to healthcare-associated infections (3, 4).

In dentistry, biofilm contamination has been most intensively investigated in dental unit waterlines (DUWLs), where warm, stagnant water and plastic tubing promote colonization and persistence (5–9). Opportunistic pathogens, such as *Legionella pneumophila* and *Pseudomonas aeruginosa,* are frequently detected in DUWLs and have been associated with infection risks, particularly among immunocompromised individuals (10–16). Additional taxa, including *Stenotrophomonas*, *Acinetobacter*, and *Klebsiella*, further complicate disinfection efforts due to their intrinsic and acquired resistance traits (13, 15–17). Dental procedures also generate aerosols capable of disseminating biofilm-associated microorganisms within clinical environments (11, 18).

By contrast, dental chair spittoons remain poorly characterized, despite continuous exposure to saliva, blood, and irrigants—conditions highly conducive to biofilm development (19, 20). The internal tubing of spittoons is difficult to access and clean, allowing microbial colonization and potential persistence over time. Each clinical department may apply distinct antibiotics and disinfectants during patient care, generating localized selective pressures that could favor the proliferation of resistant strains (19, 21). Despite extensive research on DUWL biofilms, the potential of spittoon biofilms to harbor antimicrobial resistance genes (ARGs) and mobile genetic elements remains unexplored.

Biofilms facilitate antimicrobial resistance dissemination by promoting horizontal gene transfer via plasmids, integrons, and transposons within dense microbial communities (22–24). Understanding how these processes operate in dental environments is essential for improving infection-control strategies and minimizing the dissemination of resistant microorganisms.

High-throughput sequencing enables detailed characterization of complex microbial communities, including resistome composition and mobile genetic elements. Combining 16S rRNA gene sequencing with shotgun metagenomics provides complementary insight into community structure and functional potential (25–29).

Here, we investigated dental chair spittoon biofilms across four departments of a university dental clinic. We hypothesized that spittoon biofilms harbor distinct microbial and resistome profiles shaped by department-specific environments and temporal variation. Specifically, we aimed to (i) characterize microbial diversity and community structure across departments, (ii) assess temporal shifts in biofilm composition, (iii) identify and classify antimicrobial resistance genes, including plasmid-associated determinants, and (iv) functionally validate antimicrobial resistance through culture-based phenotypic susceptibility testing of spittoon-derived isolates. By integrating community ecology, functional metagenomics, and phenotypic validation, this study provides a systems-level view of dental spittoon biofilms as structured reservoirs of antimicrobial resistance within clinical water infrastructures.

## MATERIALS AND METHODS

### Study design and setting

Biofilms were collected from dental chair spittoons across four departments of a university dental polyclinic (Oral Surgery, Periodontics, Orthodontics, and Prosthetics). Sampling was performed at three time points (September 2024, January 2025, and April 2025) to capture temporal dynamics. For each department, three high-use chairs were selected, yielding a core data set of 36 biological samples (4 departments × 3 chairs × 3 time points). Additionally, samples from additional chairs (e.g., PE6-7 in Periodontics, S6-6.1 in Oral Surgery, and D6 in Prosthetics) were included at selected time points as biological replicates, expanding the data set to a total of 53 samples. To evaluate the influence of the DNA extraction method, a subset of samples from September and January was processed in parallel with two commercial kits: the FastDNA Spin Kit for Soil (MP Biomedicals) and the ZymoBIOMICS DNA Miniprep Kit (Zymo Research). These served as technical replicates of the same biological material. Because the FastDNA kit consistently yielded higher DNA concentrations and improved library preparation efficiency, all samples from the final time point (April 2025) were extracted using the FastDNA kit to standardize downstream sequencing workflows.

### Sampling and transport

Sampling was conducted in the morning (07:30–08:15) before the start of patient care and prior to daily disinfection procedures. Operators wearing sterile nitrile gloves inserted sterile flocked swabs preloaded with eNAT medium into the spittoon drain opening and rotated them using a standardized spiral motion (~15 rotations) to scrape the wetted internal surface (~10 cm length). Three replicate swabs from the same chair and time point were pooled into a single eNAT tube to maximize DNA yield and reduce intra-chair variability. Samples were transported on wet ice (2°C–8°C) and stored at −80°C within 90 min of collection until DNA extraction.

For culture-based isolation, additional samples were collected on the same day using BD ESwabs (Becton Dickinson). These swabs were inserted into the spittoon drains and rotated to sample the internal surface as described above. ESwabs were transported at 2°C–8°C and processed for cultivation within 4 h of collection. Field blanks (one unopened eNAT tube and one air-exposed swab per sampling day) were processed in parallel and sequenced as negative controls for molecular analyses.

### DNA extraction

DNA was extracted using the FastDNA Spin Kit for Soil (MP Biomedicals) according to the manufacturer’s protocol, including mechanical lysis on a FastPrep-24 5G (MP Biomedicals) at 6.0 m/s for 2 × 40 s, with a 2-min ice cooldown between cycles. DNA was eluted in 50 µL of nuclease-free water. To evaluate extraction bias, a subset of September and January samples (*n* = 30) was processed in parallel using the ZymoBIOMICS DNA Miniprep Kit (Zymo Research). Extraction blanks accompanied each batch to monitor contamination. DNA concentration and purity were assessed with a Qubit dsDNA HS assay (Thermo Fisher Scientific) and NanoDrop One spectrophotometer; only extracts with ≥5 ng/µL and A260/280 between 1.8 and 2.1 were retained for sequencing.

### 16S rRNA gene amplicon library preparation and sequencing

The V3–V4 region of the 16S rRNA gene was amplified using primers 338F (5′-ACTCCTACGGGAGGCAGCA-3′) and 806R (5′-GGACTACHVGGGTWTCTAAT-3′). PCRs (25 µL) contained 12.5 µL 2 × KAPA HiFi HotStart ReadyMix, 0.2 µM of each primer, and 2 µL of template DNA (5–20 ng). Cycling conditions: 95°C for 5 min; 25 cycles of 95°C for 30 s, 50°C for 30 s, 72°C for 40 s; final extension at 72°C for 7 min. Duplicate PCRs were pooled, purified with AMPure XP beads (0.8× ratio), quantified by Qubit, and normalized equimolarly. Libraries were dual-indexed and sequenced on an Illumina NovaSeq 6000 (2 × 250 bp, Biomarker Technologies, Münster, Germany).

### Amplicon bioinformatics

Demultiplexed reads were processed in QIIME 2 (v2022.2) using DADA2 for filtering, trimming, denoising, merging, and chimera removal (truncLen = 240/200, trimLeft = 17/21, maxEE = 2, and chimera = “consensus”) (30, 31). Taxonomy was assigned using a SILVA 138.1 naïve Bayes classifier trained on the V3–V4 region (32). The resulting feature table, taxonomy, and metadata were imported into R (v4.2.2) via phyloseq for downstream analyses.

Putative contaminants were identified with *decontam* (prevalence method, threshold = 0.10) using field, extraction, and no-template controls; flagged ASVs were removed. Non-bacterial sequences (mitochondria and chloroplasts) were filtered prior to ecological analyses. Alpha diversity (Observed richness and Shannon index) was calculated on tables rarefied to an equal sequencing depth of 4,140 reads per sample, corresponding to the median sequencing depth of the retained FastDNA data set. This threshold was selected to maximize retention of low-biomass samples while preserving a balanced longitudinal design. To evaluate the impact of rarefaction, alpha-diversity analyses were additionally repeated on non-rarefied data.

Beta-diversity was assessed using Bray-Curtis dissimilarities on relative abundances, ordinated by PCoA, and tested by PERMANOVA (vegan::adonis2, 9,999 permutations). Homogeneity of dispersion was evaluated with *betadisper*/*anova*. Differential abundance at the genus level was analyzed using DESeq2 (design = ~Polyclinic + Month) with Benjamini-Hochberg correction (*q* < 0.10). Genera were prefiltered by prevalence ≥20% and median relative abundance ≥0.1%. Non-significant results were masked in heatmaps, and log₂ fold changes were truncated at ±3. Separate DESeq2 contrasts were also performed among sampling months (September 2024, January 2025, and April 2025) to capture temporal shifts in genus-level abundances associated with biofilm maturation.

### Community composition and core microbiome analysis

Amplicon sequence variants (ASVs) were agglomerated to the genus level using *tax_glom* (phyloseq v1.46). Relative abundances were calculated per sample, excluding features with prevalence <20% or median relative abundance <0.1%. The core microbiome was defined as genera present in ≥10% of samples within each department at ≥0.05% relative abundance, representing the intersection of departmental cores. To assess the sensitivity of this definition, additional analyses were performed using stricter prevalence thresholds of 50% and 80% while maintaining the same relative-abundance cutoff. Core and unique genera were visualized using ComplexUpset (v1.3).

### Shotgun metagenomic sequencing

A subset of nine high-biomass samples (≥25 ng/µL DNA, high 16S richness) was selected for shotgun metagenomic sequencing. Libraries were prepared using the Nextera DNA Flex kit (Illumina), size-selected for ~350 bp inserts, and sequenced on an Illumina NovaSeq PE150 platform (~1 Gb per sample). Reads were uploaded to Kbase (33) and quality-checked with FastQC and trimmed with Trimmomatic (v0.39) (34, 35). Assemblies were generated with metaSPAdes (36), retaining contigs ≥1 kb. Post-assembly quality metrics, including N50, total contig length, and GC content, were assessed using QUAST.

### Metagenome-assembled genomes and annotation

Contigs were binned using MaxBin2 (37), MetaBAT2 (38), and CONCOCT (39), with consensus bins selected via DASTool (40). Genome completeness and contamination were evaluated using CheckM (41). Bins meeting MIMAG medium-quality criteria (>50% completeness, <10% contamination) were retained. Taxonomic classification was performed with GTDB-Tk (42) and functional annotation with DRAM (43), focusing on carbon utilization, energy metabolism, membrane transport, and nitrogen cycling modules.

### Resistome and plasmid profiling

ARGs were identified using AMRFinderPlus (44) and cross-validated with Abricate (ResFinder database; ≥90% identity, ≥60% coverage) (45). Detected ARGs were categorized by antibiotic class and normalized as gene counts per Mb of assembly. Contigs were screened with PlasmidFinder (Galaxy Version 2.1.6) (46) for plasmid replicons. To assess potential physical linkage between ARGs and plasmid-associated elements, contigs were screened for the co-occurrence of ARGs and plasmid replicon markers. Contigs carrying both features on the same assembly were considered putative mobilizable elements.

AMRFinderPlus outputs were merged across departments. For each sample, ARG richness was defined as the number of unique gene symbols per sample. Class-level composition was derived from the *Class* field as the percentage of all ARG hits per department. Gene exemplars cited in the text represent the most frequent entries within each antibiotic class. The association between 16S Shannon diversity and ARG richness was tested by Spearman correlation using the nine paired September 2024 samples.

### Culture-based isolation, identification, and antimicrobial susceptibility testing

Biofilm material collected from dental chair spittoons was cultured on selective and differential media to recover both susceptible and antimicrobial-resistant microorganisms. Samples were plated on Columbia blood agar supplemented with 5% sheep blood, MacConkey agar, CHROMagar ESBL, CHROMagar MRSA bi-plates, BCYE agar for *Legionella* spp., and CHROMagar Candida. Plates were incubated aerobically at 37°C–42°C for 24–48 h unless otherwise specified; BCYE plates were incubated at 37°C. Anaerobic cultivation was performed using BD GasPak EZ systems or anaerobic chambers.

For non-selective and selective enrichment, aliquots were incubated in Tryptic Soy Broth (TSB), Brain Heart Infusion (BHI), or LY-BHI under aerobic or anaerobic conditions, followed by subculture onto solid media. In selected experiments, antibiotic-supplemented TSB containing third-generation cephalosporins was used to enhance recovery of resistant strains. Up to four morphologically distinct colonies per plate were subcultured to ensure purity and stored at −80°C in glycerol-containing medium.

Isolates were identified to species level by MALDI-TOF mass spectrometry (VITEK MS PRIME, bioMérieux). Antimicrobial susceptibility testing (AST) was performed using the VITEK 2 automated system (bioMérieux), and minimum inhibitory concentrations (MICs) were interpreted according to EUCAST clinical breakpoints (version 15.0). Results were categorized as S (susceptible, standard dosing regimen), I (susceptible, increased exposure), R (resistant), or insufficient evidence (IE) according to EUCAST definitions. Where applicable, extended resistance screening was performed using Micronaut S MDR MRGN-Screening 3 panels (MERLIN Diagnostika).

### Statistical analysis and visualization

Alpha-diversity metrics were compared using Kruskal–Wallis tests followed by Benjamini-Hochberg-corrected Wilcoxon tests. Beta-diversity was tested with PERMANOVA (adonis2, 9,999 permutations, strata = chair_id). Differential abundance of ARG classes was evaluated with ALDEx2 after centered log-ratio transformation. To assess the relative contributions of stochastic and deterministic processes, a null-model analysis based on the Raup-Crick metric (RCbray) was implemented using the vegan package. Pairwise RCbray values were used to quantify community assembly mechanisms, with |RCbray| ≤ 0.95 indicating stochastic (neutral) assembly.

Figures were generated using ggplot2 (v3.5.1), phyloseq (v1.42.0), and ComplexHeatmap. All analyses were performed in R (v4.2.2).

## RESULTS

### Alpha and beta diversity of spittoon biofilms across departments

**Fig 1:**
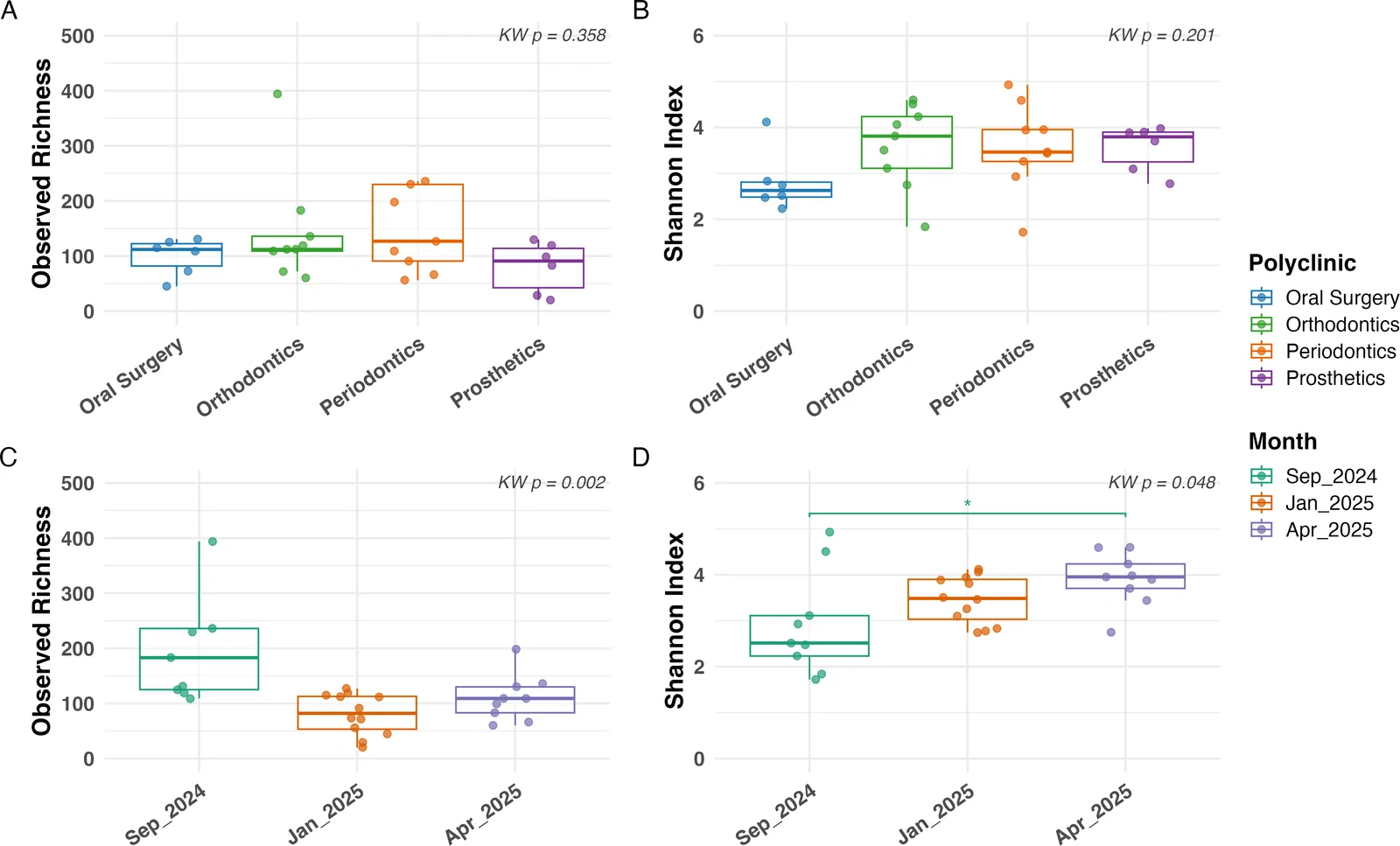
Alpha diversity of spittoon biofilms. (**A**) Observed richness and (**B**) Shannon index by department. (**C–D**) The same metrics by sampling month (September 2024, January 2025, and April 2025). Boxes show median and interquartile range; whiskers extend 1.5× IQR; points represent individual samples. KW *P* value results from overall group differences among departments or sampling months.

To assess whether microbial diversity differed among departments and over time, alpha diversity metrics were calculated from samples extracted with the FastDNA kit. Both richness and evenness varied across departments, with the highest median diversity in *Periodontics* and *Prosthetics* and the lowest in *Oral Surgery* (Fig. 1A and B). However, none of the department-level differences reached statistical significance (Kruskal-Wallis: Shannon *P* = 0.201; Observed *P* = 0.358) (Table S1). Median Shannon values ranged from 2.6 to 3.9, and observed richness from ~80 to 130 ASVs per sample.

To assess the robustness of diversity estimates, we examined sequencing-depth distributions and repeated alpha-diversity analyses on non-rarefied data. Sequencing depth varied substantially among samples (min = 530, Q1 = 2,626, median = 4139.5, mean = 65,873, Q3 = 121,433, and max = 362,031 reads per sample). Rarefaction curves indicated that the selected threshold captured the most common taxa while retaining low-biomass samples (Fig. S1). Reanalysis of alpha diversity using non-rarefied data produced the same overall interpretation, with no significant department-level differences and significant month-associated differences for both Shannon diversity and observed richness.

In contrast, the sampling month significantly affected alpha diversity, with both Shannon (*χ*² =6.07, *P* = 0.048) and observed richness (*χ*² =12.36, *P* = 0.0021) differing among months (Fig. 1C and D; Table S1). Diversity increased from September 2024 to April 2025, suggesting temporal enrichment and evenness of the biofilm community. The observed richness declined from September 2024 to January 2025. Other metrics (Chao1 and Pielou) showed consistent trends (Fig. S2).

**Fig 2:**
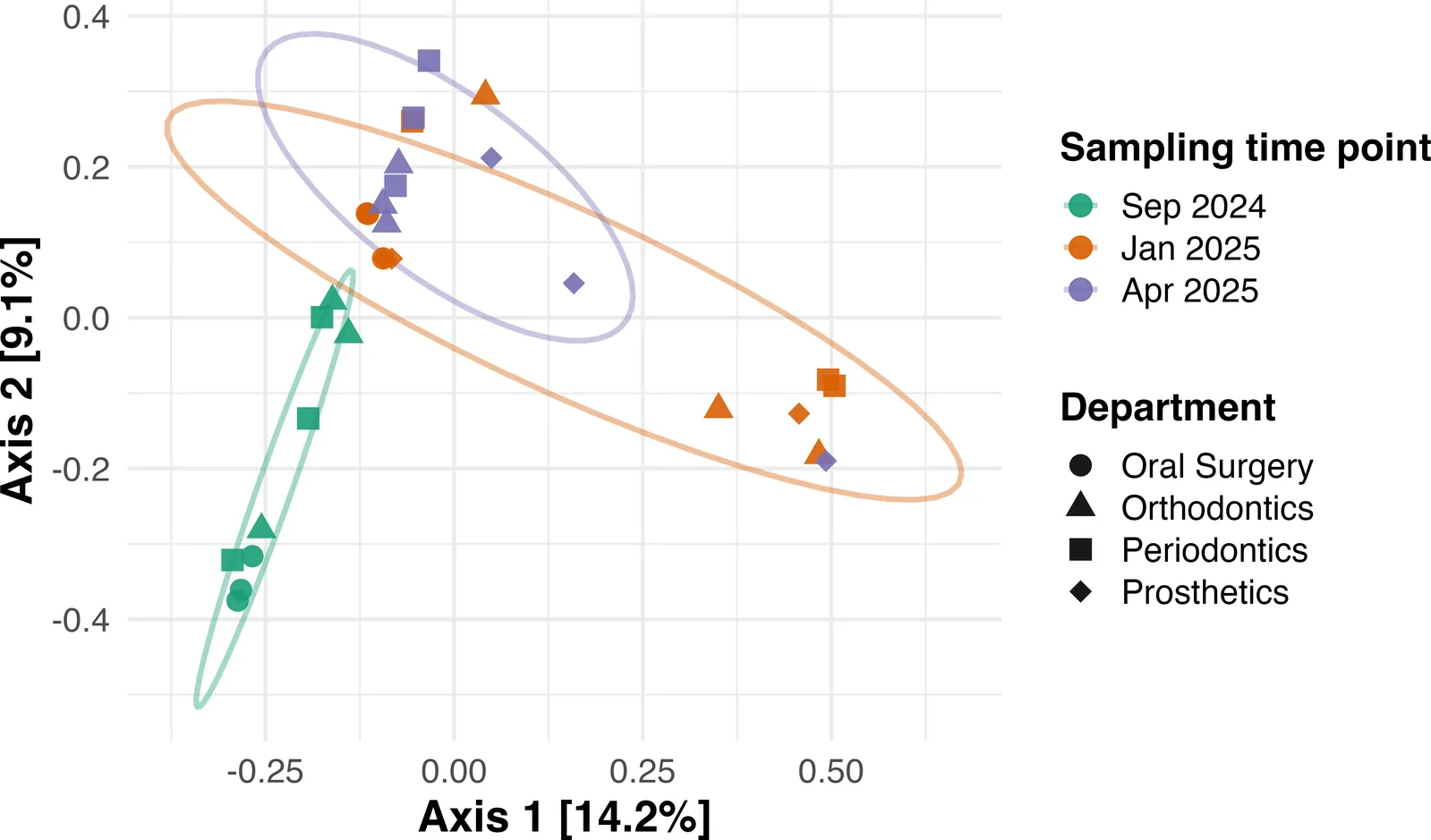
PCoA of Bray-Curtis dissimilarities. Points are colored by department and shaped by sampling time points. Ellipses denote 80% confidence areas for each month. Axis 1 and Axis 2 explain 14.2% and 9.1 of the variance.

Principal coordinate analysis (PCoA) of Bray-Curtis dissimilarities revealed that samples clustered primarily by sampling time point, with weaker separation by department (Fig. 2; Fig. S3 and Table S2).

### Effect of the DNA extraction method

To evaluate methodological bias, a subset of samples from September 2024 and January 2025 was re-extracted using two DNA isolation kits (FastDNA and ZymoBIOMICS). Shannon diversity did not differ between kits (Wilcoxon *P* = 0.091; Welch *t* = 0.154; Fig. S4), although PERMANOVA detected a small but significant kit effect (*R*² =0.039, *P* = 0.018) (Table S3). Together, these analyses show that biological factors (department and time) dominate variation, with only minor contributions from extraction chemistry.

### Microbial community composition

**Fig 3:**
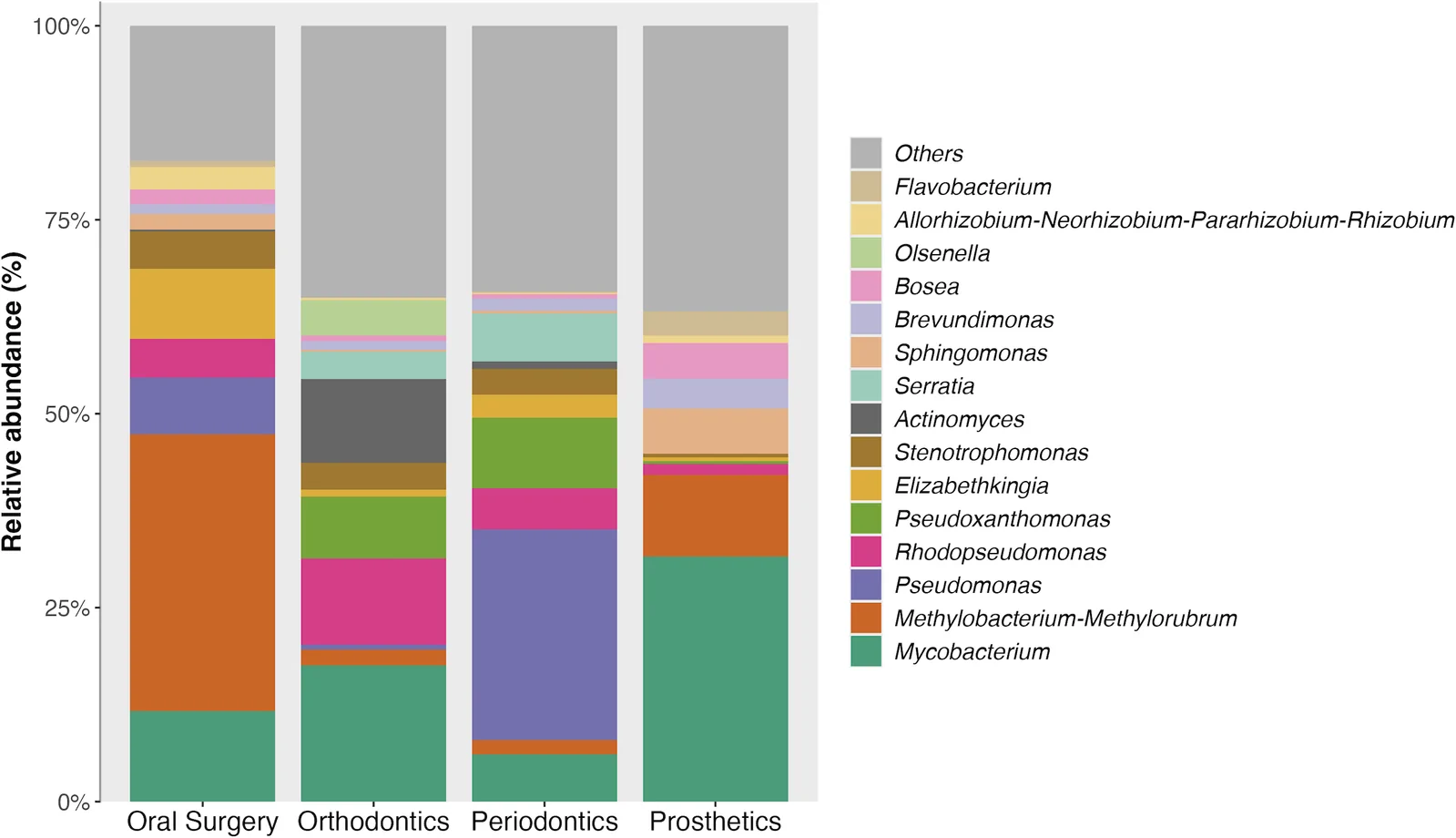
Relative abundance of dominant genera across departments. Stacked bar plot showing the 15 most abundant genera in spittoon biofilms from four dental departments.

Genus-level profiles revealed distinct community signatures among departments (Fig. 3). Prosthetic unit biofilms were dominated by water-associated taxa such as *Mycobacterium* and *Methylobacterium–Methylorubrum*, with smaller fractions of the opportunistic pathogen *Pseudomonas* and *Elizabethkingia,* an emerging pathogen in hospital environments. Oral Surgery showed a mixed profile, combining *Mycobacterium*, *Pseudomonas*, and *Elizabethkingia*, together with minor contributions from *Brevundimonas* and *Bosea*. Orthodontics was characterized by higher relative abundances of *Rhodopseudomonas*, *Bosea*, and *Sphingomonas*, while Periodontics was enriched in *Rhodopseudomonas* and *Elizabethkingia*, along with *Actinomyces* and *Mycobacterium*. Across departments, the most consistently abundant genera included *Mycobacterium, Methylobacterium–Methylorubrum, Bosea,* and *Elizabethkingia*, together with intermittent oral commensals (*Actinomyces* and *Prevotella*) and accounted for ~65%–80% of total reads.

**Fig 4:**
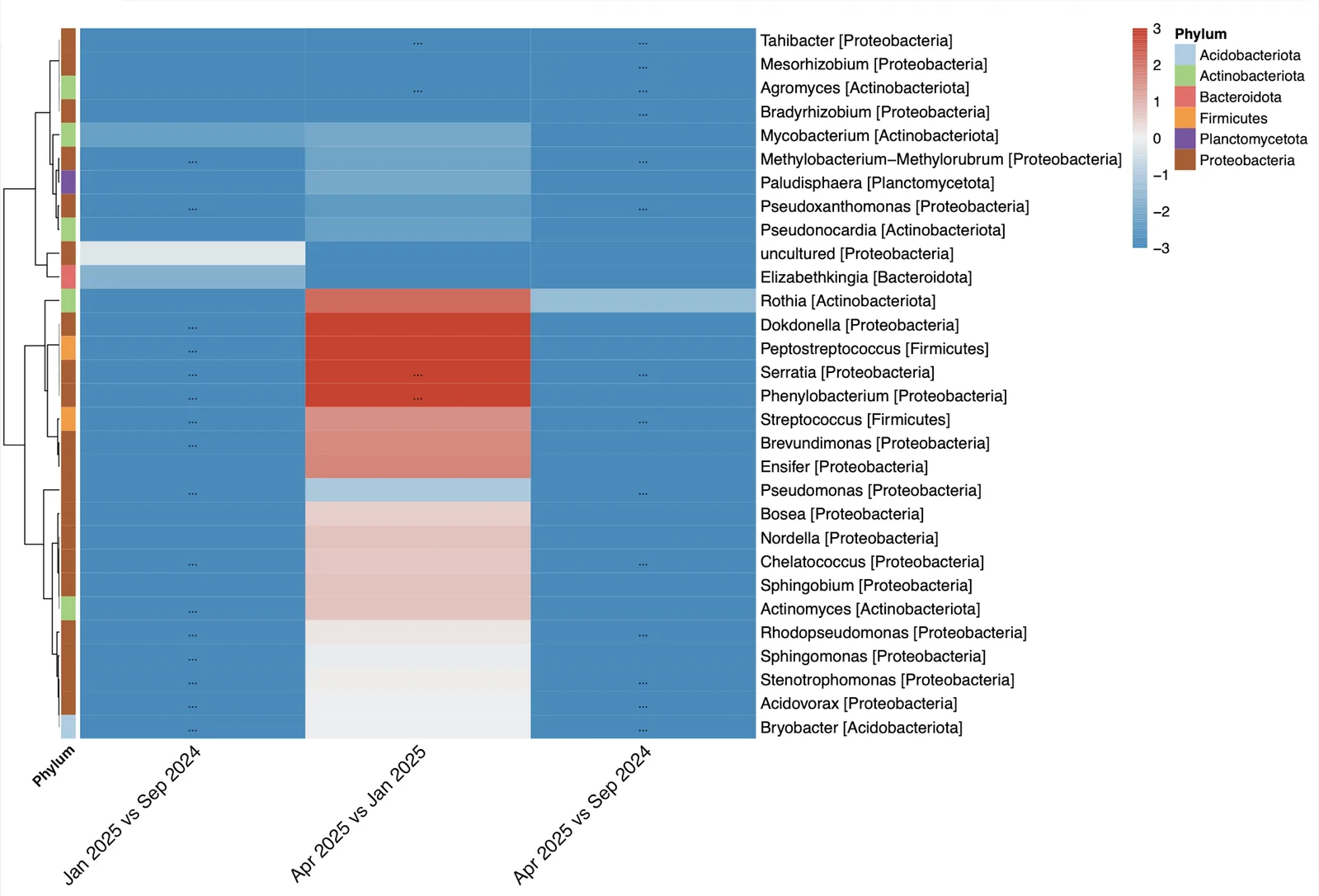
Differentially abundant genera across sampling periods (DESeq2, *q* < 0.10). Heatmap of log₂ fold-change contrasts among months shows progressive enrichment of water-associated taxa and decline of oral commensals over time. Red indicates enrichment and blue depletion relative to the reference period.

Differential-abundance analysis (DESeq2; *q* < 0.10) identified several genera that were significantly enriched by both department and sampling period (Fig. 4; Fig. S5). While department-level contrasts highlighted spatial differentiation, a stronger temporal signal emerged across sampling months (September 2024, January 2025, and April 2025). Temporal DESeq2 comparisons revealed progressive enrichment of water-associated genera such as *Methylobacterium–Methylorubrum*, *Bradyrhizobium*, *Paludisphaera*, and *Pseudoxanthomonas* between January and April 2025, accompanied by shifts in oral-associated genera including *Rothia*, *Streptococcus*, and *Actinomyces* (Fig. S5). The transition from September to January was marked by increased *Elizabethkingia* and *Brevundimonas*, followed by the later dominance of *Mycobacterium* and *Methylobacterium* lineages.

Using the original prevalence threshold (10%), 12 genera formed a shared genus set across all departments (Table S4). Water-associated taxa, including *Methylobacterium–Methylorubrum*, *Mycobacterium*, *Elizabethkingia*, *Bosea*, and *Sphingomonas,* dominated this set. Sensitivity analyses using stricter prevalence thresholds reduced the shared set to three genera at 50% prevalence (*Methylobacterium–Methylorubrum*, *Bosea*, and *Sphingomonas*) and no genera at 80% prevalence. These results indicate that the broader definition captures low-prevalence taxa shared among departments, whereas the strict core is restricted to a small number of ubiquitous water-associated biofilm genera.

Additionally, Indicator-taxon analysis (IndVal.g) identified statistically significant department-specific genera consistent with DESeq2 patterns, such as *Prevotella* and *Actinomyces* (Periodontics), *Pseudomonas* and *Stenotrophomonas* (Oral Surgery), and *Mycobacterium* (Prosthetics) (Table S5).

Null-model analysis using the Raup-Crick metric showed that most pairwise community comparisons fell within the stochastic range (|RCbray| ≤ 0.95; median RCbray ≈ 0.01), indicating that neutral processes such as dispersal and ecological drift dominated spittoon biofilm assembly (Fig. S6). Only a small fraction of comparisons (max = 1.0) deviated toward deterministic selection, consistent with a secondary contribution of ecological filtering during later sampling periods. Thus, the data support predominantly stochastic assembly with modest evidence of increased ecological filtering over time, rather than a complete transition to deterministic assembly.

Collectively, these results highlight that spittoon biofilms harbor a stable but compositionally distinct microbiota shaped by local environmental conditions and clinical activity and seasonal change.

### Shotgun metagenomic sequence data

A subset of nine high-biomass spittoon biofilm samples, representing Orthodontics, Periodontics, and Oral Surgery, were subjected to shotgun metagenomic sequencing, generating a total of ≈203 million paired-end reads corresponding to ≈60.7 Gb of clean data, with an average yield of 6.7 Gb per sample. Quality metrics were consistently high (>97% bases ≥ Q20, >91% ≥Q30, GC content = 51%–63%). Following assembly, nine medium- to high-quality metagenome-assembled genomes (MAGs) were recovered (CheckM completeness = 72%–98%, contamination <3.5%). These MAGs represented the dominant, water-adapted taxa within spittoon biofilms and were used for DRAM-based functional annotation. This targeted subset was selected to maximize metagenome assembly quality and enable detailed resistome characterization rather than to provide a comprehensive survey of all departments, sampling periods, and biofilm developmental stages represented in the full cohort. This enrichment strategy may preferentially capture mature or heavily colonized biofilms and therefore may not fully represent the broader diversity of spittoon biofilms sampled throughout the study.

### Metagenomic insights into antimicrobial resistance

Metagenomic sequencing revealed a complex resistome comprising 29 non-redundant ARGs spanning seven antibiotic classes, reflecting a diverse functional repertoire within these clinical biofilms.

**Fig 5:**
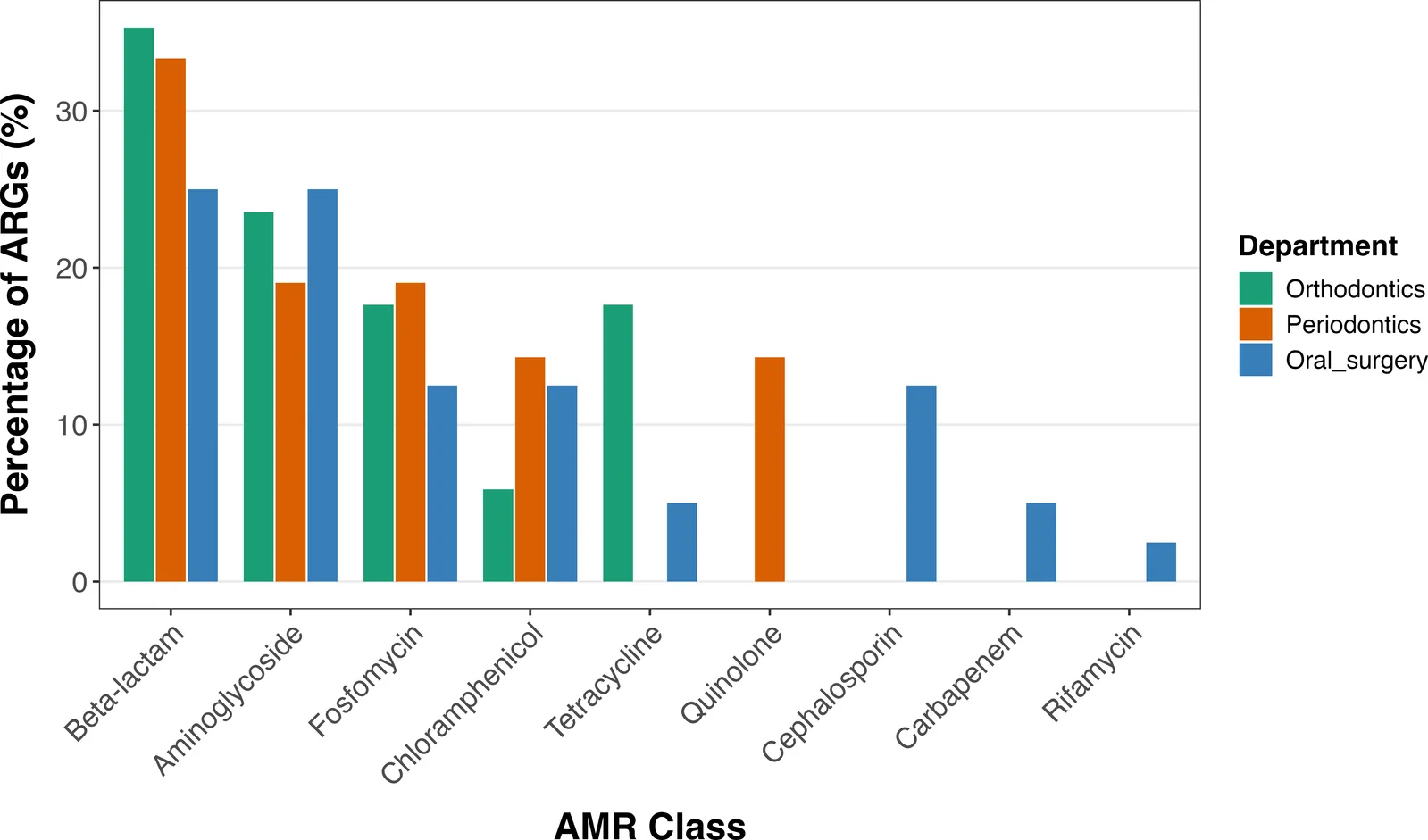
Distribution of ARG classes across departments. Bar plots show the relative proportion of ARGs by antibiotic class in metagenomic assemblies from Orthodontics, Periodontics, and Oral Surgery biofilms.

Across departments, β-lactam resistance genes dominated, followed by aminoglycoside and fosfomycin (Fig. 5; Table S7A). ARG richness (unique genes per sample) ranged from 4 to 22, with median values of 4 in Orthodontics, six in Periodontics, and eight in Oral Surgery.

**Fig 6:**
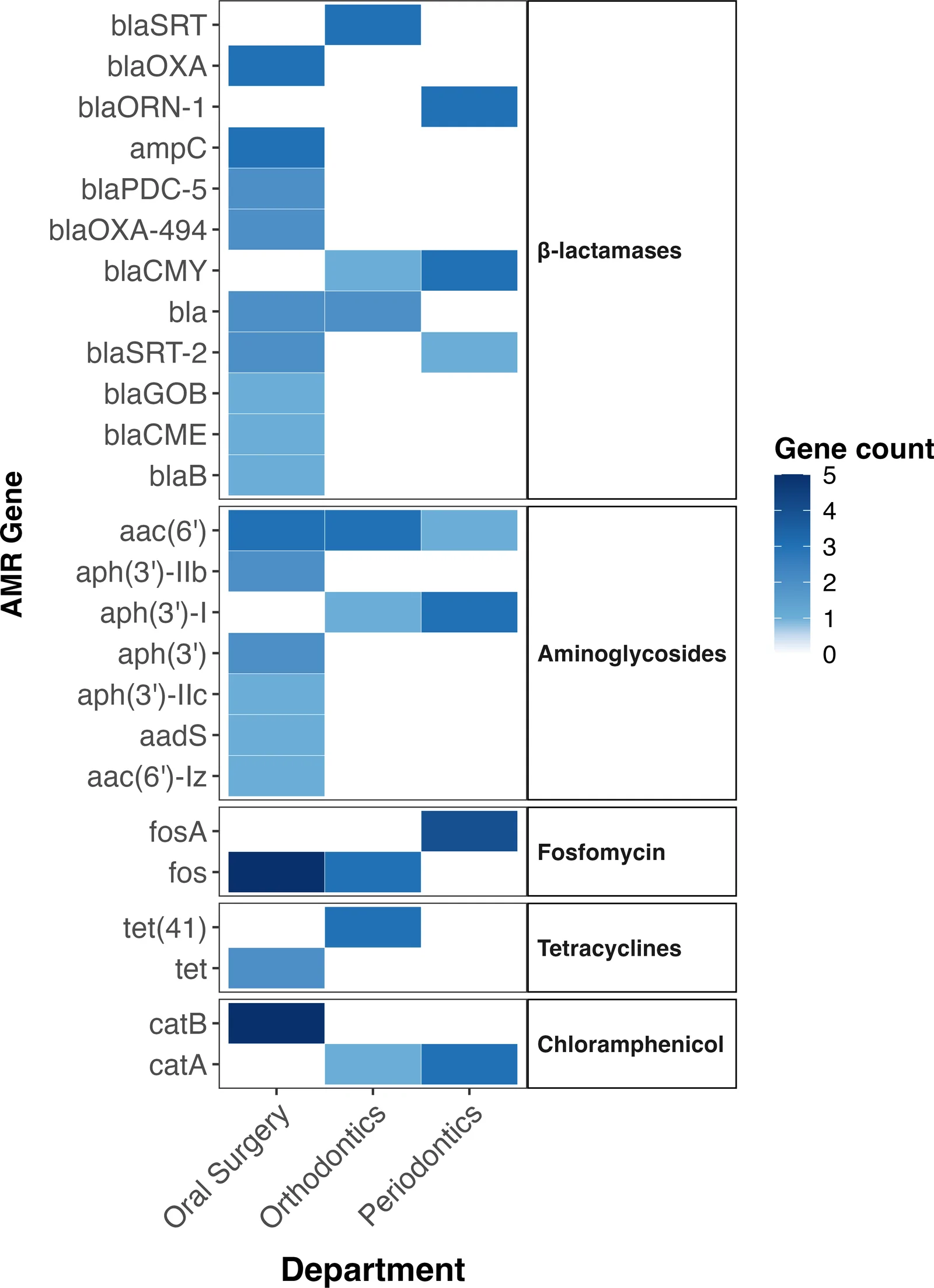
Heatmap showing AMR gene abundance by department, grouped by resistance class. Blue gradient reflects gene counts (0–5).

At the gene level, department-specific patterns were evident. In Oral Surgery, the most frequently detected ARGs were *fos* (*n* = 5) and *catB* (*n* = 5), together with *aac(6′*), *bla_OXA_*, and *ampC* (each *n* = 3) (Fig. 6). Additional β-lactamase variants and aminoglycoside-modifying enzymes occurred at lower frequencies. Orthodontic biofilms were characterized by *aac(6*′), *blaSRT*, *fos*, and *tet(41*) (all *n* = 3), along with single detections of *blaCMY*, *catA*, and *aph(3′)-I*. In Periodontics, *fosA* (*n* = 4) was dominant, accompanied by *blaCMY*, *blaORN-1*, *catA*, and *aph(3′)-I* (each *n* = 3) and the efflux pump marker *oqxB* (*n* = 3) (Fig. 6; Table S7B).

Screening of metagenomic assemblies with PlasmidFinder identified several plasmid replicon types, including *IncFII(Yp*), *IncFIB(K*), *Col440I*, and *RepA*, predominantly in orthodontic (O1–O2) and periodontic (PE1–PE3) samples. Although ARGs and replicon markers were not co-localized on the same contigs, all plasmid-positive samples also contained multiple ARG-bearing bins (4–22 unique genes per sample; Table S8). This co-occurrence indicates that mobile genetic elements and resistance determinants coexist within the biofilm networks, potentially enabling horizontal gene transfer under favorable ecological conditions.

Beyond the resistome, functional annotation of MAGs with DRAM revealed a conserved suite of major metabolic pathways across all departments (Fig. S7 and S8).

### Integration of taxonomic and resistome profiles

Integration of 16S and metagenomic data sets revealed a negative association between bacterial diversity and resistome complexity. Shannon diversity was inversely correlated with ARG richness (Spearman *r* = −0.85, *P* = 0.0035; Fig. S9), indicating that more diverse microbial communities were associated with lower ARG richness. Departments dominated by opportunistic pathogens such as *Elizabethkingia*, *Pseudomonas*, and the water-associated *Mycobacterium* exhibited the highest abundance of β-lactamase and tetracycline resistance genes.

### Phenotypic confirmation of antimicrobial resistance in spittoon-derived isolates

A total of 162 culturable bacterial isolates were recovered from dental spittoon biofilms and taxonomically identified using MALDI-TOF MS/VITEK (Table S9). Phenotypic antimicrobial susceptibility testing revealed that resistance traits were expressed by a subset of viable biofilm-associated bacteria. Overall, 21.7% of isolates showed resistance to at least one antibiotic, while 10.6% exhibited resistance to two or more agents, and 5.0% fulfilled the criteria for multidrug resistance (MDR), defined as resistance to three or more antimicrobial classes.

**Fig 7:**
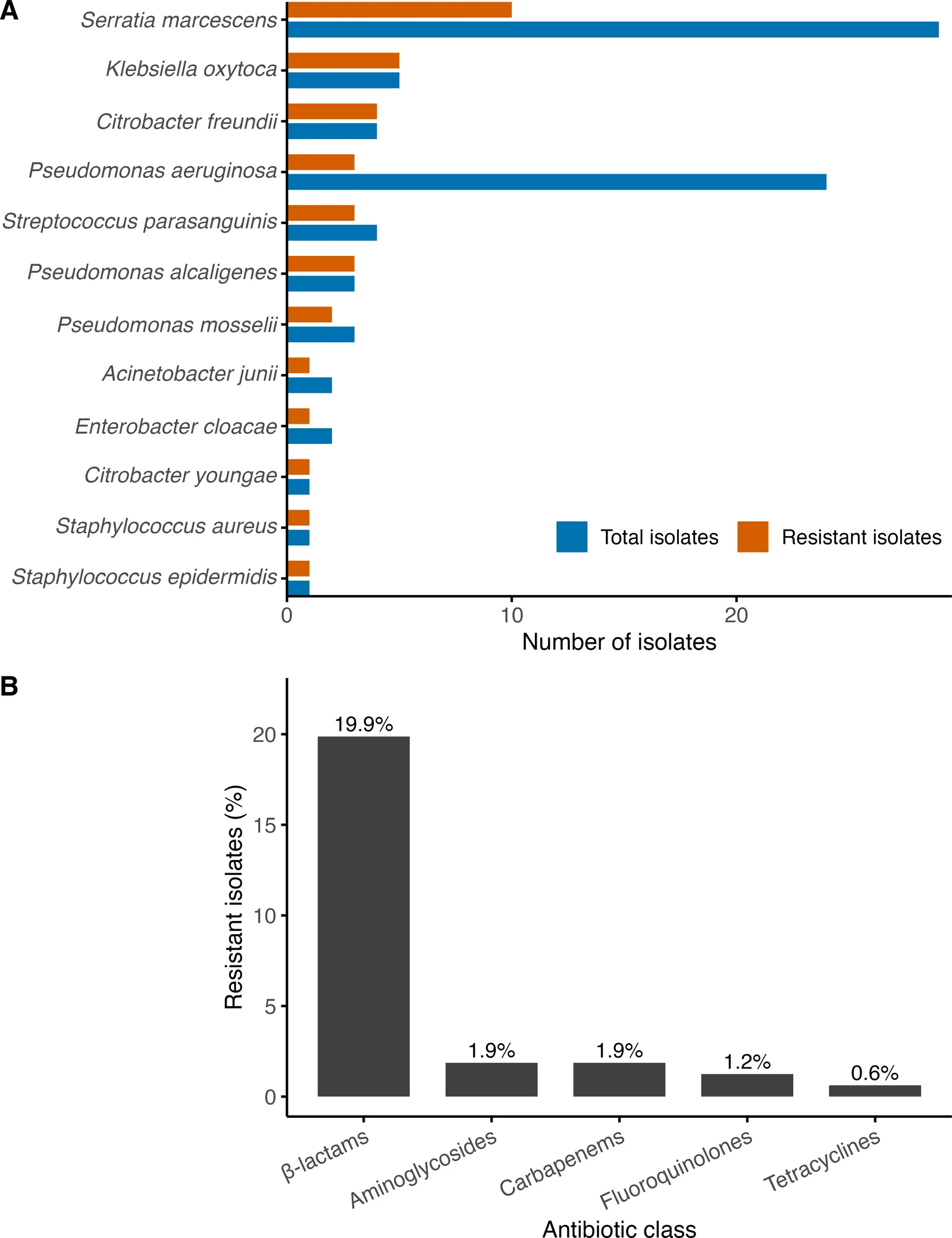
Phenotypic antimicrobial resistance patterns among spittoon-derived isolates. (**A**) Distribution of total and resistant isolates across bacterial species recovered from dental spittoon biofilms. Only taxa with at least one resistant isolate are shown. (**B**) Proportion of isolates resistant to at least one agent within each antibiotic class, expressed as percentage of the total tested isolates (*n* = 162). Resistance interpretations were based on EUCAST clinical breakpoints.

Multidrug-resistant phenotypes were predominantly associated with gram-negative, water- and environment-adapted opportunistic pathogens, reflecting the selective pressures imposed by spittoon biofilm habitats. MDR isolates were frequently recovered among members of the Enterobacterales, including *Klebsiella pneumoniae, Klebsiella oxytoca, Enterobacter cloacae* complex, *Citrobacter* spp., and *Serratia marcescens*, as well as among non-fermenting gram-negative taxa such as *Pseudomonas* spp., *Acinetobacter* spp., *Achromobacter* spp., and *Stenotrophomonas maltophilia*. However, multi-resistant *Escherichia coli* were also identified. Within these groups, resistance commonly spanned multiple antibiotic classes, indicating broad phenotypic resistance profiles rather than isolated single-drug resistance (Fig. 7A).

When summarized at the level of antibiotic classes, resistance was most frequently observed against β-lactams (19.9%), whereas resistance to fluoroquinolones (1.2%), aminoglycosides (1.2%), and tetracyclines (0.6%) was comparatively infrequent (Fig. 7). These class-level patterns align with the dominance of β-lactam resistance genes detected in metagenomic resistome analyses.

Notably, resistance to carbapenems was detected in a small number of isolates, representing the most clinically relevant resistance phenotype observed in this culture-based analysis. Three isolates—two *Pseudomonas* spp. and one *Acinetobacter junii*—exhibited resistance to imipenem and/or meropenem, often in combination with resistance to additional antibiotic classes. Although rare, the isolation of carbapenem-resistant phenotypes without employing pre-enrichment procedures underscores the capacity of dental spittoon biofilms to sustain viable bacteria expressing resistance to last-line antimicrobials.

## DISCUSSION

### Ecological dynamics of spittoon biofilms

This study provides an ecological and resistome characterization of dental spittoon biofilms, revealing structured, resilient microbial communities shaped by the unique interface between patient-generated fluids and aerosols, water exposure, and intermittent chemical disinfection. Across three sampling periods, we observed a progressive increase in Shannon diversity and richness. The reason for this is not yet clear; however, seasonal influences on the composition of biofilms in drains have been described.

Comparable temporal dynamics have been reported in DUWL systems, where longitudinal monitoring reveals communities that are stable yet adaptable, responding more strongly to operational conditions than to clinical specialization (20). The absence of significant differences in multivariate dispersion further suggests that temporal change reflects compositional turnover rather than altered heterogeneity.

Community differentiation by department and the significant department × time interaction may reflect localized environmental differences among departments. Potential contributing factors include variation in patient turnover, irrigant use, aerosol exposure, and disinfection practices, although these variables were not directly quantified in the present study. These findings align with observations in DUWLs, where operational variables such as water stagnation, tubing material, flushing frequency, and disinfectant regime shape biofilm structure and resilience (5, 20).

Principal coordinate analysis confirmed that β-diversity was primarily structured by sampling time, with weaker separation among departments and a significant interaction between department and time, indicating context-dependent community shifts.

### Spittoons as a distinct ecological niche

At the community level, spittoon biofilms displayed a hybrid oral-environmental signature. While oral-associated genera such as *Actinomyces*, *Streptococcus*, and *Rothia* were consistently detected, they occurred predominantly and at low relative abundance, suggesting transient colonization by saliva-derived microorganisms (47–49).

In contrast, the dominant taxa—including *Sphingomonas*, *Mycobacterium*, *Elizabethkingia*, *Methylobacterium–Methylorubrum*, and *Bosea*—represent water-adapted, disinfectant-tolerant lineages well documented in healthcare and dental water systems (50–56). Sensitivity analyses demonstrated that only three water-associated genera (*Methylobacterium–Methylorubrum*, *Bosea*, and *Sphingomonas*) remained shared across departments under a 50% prevalence threshold, whereas no genera met an 80% threshold. This suggests that much of the broader shared genus set reflects low-frequency taxa occurring across multiple departments.

The temporal enrichment of *Mycobacterium*, *Methylobacterium*, *Bradyrhizobium*, and *Pseudoxanthomonas*, accompanied by the decline of oral commensals, is consistent with a directional shift from saliva-contaminated surfaces to mature aquatic biofilms adapted to oxidative and nutrient-limited conditions (54, 57).

Occasional detection of facultative pathogens such as *Serratia*, *Stenotrophomonas*, and *Elizabethkingia* may reflect opportunistic growth under fluctuating moisture conditions and exposure to disinfectants (50, 58, 59).

In practical terms, departments differed in their clinical activities and patterns of chair utilization. Such differences could influence water stagnation, organic input, and exposure to disinfectants within spittoon systems. However, these variables were not quantitatively assessed and should therefore be considered as plausible explanatory factors rather than demonstrated drivers of community variation. Orthodontics and Prosthetics typically involve longer appointments with minimal aerosol generation, whereas Oral Surgery and Periodontics produce higher volumes of splatter, irrigants, and biological fluids (60–62). Disinfectant protocols also varied, with some units using higher concentrations of quaternary ammonium or peroxide-based agents (63, 64). These operational factors are plausible contributors to differences in water stagnation, organic input, and disinfectant exposure within spittoons, although their effects were not directly quantified in this study. Consequently, department-specific community signatures likely reflect local microenvironmental heterogeneity rather than inherent biogeographic isolation (17, 65).

Although disinfectant concentrations were not quantitatively assessed in this study, the enrichment of genera previously reported to be disinfectant-tolerant is consistent with a potential role for chemical stress in community assembly.

### Biofilm resilience and functional redundancy

The recovery of medium- to high-quality MAGs across departments, combined with DRAM annotations revealing conserved central metabolism, electron transport systems, and membrane transport modules, indicates functional redundancy and metabolic versatility—hallmarks of mature, stress-tolerant biofilms. Such redundancy has been documented in DUWLs (10, 17), hospital facilities (66), and drinking water distribution systems (21).

Subtle department-level differences in nitrogen and carbohydrate metabolism suggest localized microenvironmental specialization. Variations in patient treatment profiles and irrigant chemistry may contribute to these patterns, although these factors were not directly measured. These findings support the emerging view that healthcare water-associated biofilms are not uniform, but reflect internal biogeography shaped by clinical workflows.

### Resistome structure and AMR ecology

Despite numerous studies having examined the microbial contamination of DUWLs, there is still limited research on ARGs or resistant bacterial strains (67). Moreover, each clinic may use specific antibiotics and disinfectants, selecting resistant strains *in situ* (19, 21). Such variability contributes to divergent resistome profiles and complicates infection control protocols.

Metagenomic profiling revealed a multi-class resistome dominated by β-lactamases, aminoglycoside-modifying enzymes, and fosfomycin resistance determinants. This profile mirrors resistance patterns previously observed in DUWLs and healthcare water systems, where biofilm-forming environmental opportunists act as persistent AMR reservoirs (5, 10, 13, 21, 66, 68). Consistent with reports from hospital plumbing and sink drains, taxa such as *Pseudomonas*, *Stenotrophomonas*, *Mycobacterium*, and *Elizabethkingia*–detected here as core constituents–possess well-documented intrinsic and acquired resistance to β-lactams and aminoglycosides and have repeatedly been implicated in water-associated outbreaks and AMR transmission in clinical environments (69–72). In contrast to oral plaque and periodontal microbiome studies, where tetracycline and macrolide resistance frequently dominate due to host antibiotic history and selective pressures within oral niches (49, 73), tetracycline determinants were comparatively infrequent in our data set. This divergence underscores that the spittoon microbiome is not simply an extension of the oral cavity, but rather a distinct aquatic biofilm ecosystem shaped by disinfectant exposure, intermittent water flow, and patient material. The prominence of *fosA*, consistent with findings in DUWL-associated *Pseudomonadales* and chlorine-tolerant microbes (74, 75), further supports the role of oxidative and chemical stress rather than direct antibiotic selection as a key ecological driver in this niche (76, 77).

An important limitation of the shotgun metagenomic component is the restricted size and targeted nature of the sequenced subset. Only nine high-biomass biofilm samples from three clinical departments were selected for shotgun sequencing to maximize assembly quality and resistome recovery. Notably, Prosthetics was not represented in the shotgun subset. Consequently, the identified ARG profiles, plasmid replicons, and MAG-associated functions should be interpreted as representative examples of resistance gene diversity within mature spittoon biofilms rather than comprehensive estimates of ARG prevalence across all departments, sampling periods, or biofilm developmental stages.

A notable ecological pattern was the inverse association between ARG richness and microbial diversity, suggesting that low-diversity, stress-adapted communities favor ARG accumulation—a trend consistent with studies of disinfected drinking-water networks, hospital wastewater pipelines, and industrial biofilms under oxidative pressure (1, 78–80). Such environments promote ecological filtering toward metabolically versatile, stress-tolerant specialists with intrinsic or horizontally acquired resistance traits (81).

Together, these findings support the emerging view that dental spittoons represent ecologically mature, disinfectant-conditioned biofilms that harbor relevant opportunistic pathogens and a distinct resistome architecture, functionally and compositionally separate from both oral communities and conventional DUWL systems. These findings suggest that spittoon biofilms may represent overlooked but clinically relevant reservoirs of AMR within dental healthcare environments.

### Mobile genetic elements and potential for ARG dissemination

Replicons belonging to clinically relevant incompatibility groups (IncFII, IncFIB, Col440I, and RepA) were detected in orthodontic and periodontic spittoon biofilms. Although short-read assemblies did not resolve physical linkage between ARGs and plasmid backbones, their concurrent detection within the same metagenomic samples indicates that mobile genetic elements associated with clinically important lineages are present in these systems.

Col440I replicons have been reported in association with colistin-resistant *Acinetobacter* and Enterobacteriaceae (82–84)*,* while IncFIB variants are frequently linked to carbapenemase dissemination. The detection of these plasmid backbones within stable biofilm communities demonstrates the presence of mobile genetic elements previously associated with clinically relevant antimicrobial resistance lineages. However, their occurrence in the present data set should be interpreted as evidence of co-occurrence rather than direct evidence of ARG mobilization or transmission.

These findings parallel observations in DUWL biofilms (13) and hospital water systems, where plasmid-mediated AMR has been traced to environmental persistence and biofilm-associated exchange (23, 67). While definitive assessment of ARG-plasmid linkage and transfer dynamics would require long-read sequencing, hybrid assemblies, or targeted mobilization assays, the consistent recovery of clinically associated plasmid replicons indicates that mobile genetic elements occur within these biofilm communities. These observations support the value of spittoon biofilms for environmental AMR surveillance but do not demonstrate active horizontal transfer or plasmid-mediated dissemination of resistance genes.

Importantly, ARGs and plasmid replicons were not physically linked on the same contigs; therefore, conclusions regarding mobilization potential remain speculative.

### Phenotypic confirmation of resistome potential

While metagenomic analyses revealed a multi-class resistome dominated by β-lactamases and aminoglycoside-modifying enzymes, culture-based susceptibility testing provided functional confirmation that a subset of viable biofilm-associated bacteria actively expressed these resistance traits. Among 162 tested isolates, 21.7% exhibited phenotypic resistance to at least one antimicrobial agent, and 5.0% fulfilled criteria for multidrug resistance. Because shotgun metagenomics characterized the community resistome, whereas susceptibility testing was performed on a subset of cultivable isolates, the two approaches provide complementary rather than directly comparable measures of antimicrobial resistance.

Resistance was most frequently observed among members of Enterobacterales and non-fermenting gram-negative genera, including *Serratia marcescens*, *Klebsiella oxytoca*, *Citrobacter freundii*, *Pseudomonas aeruginosa*, and *Acinetobacter junii*. The predominance of β-lactam resistance at the phenotypic level aligns with the metagenomic detection of diverse β-lactamase determinants, supporting the functional relevance of the sequencing-based resistome profile. However, direct genotype–phenotype relationships could not be established because resistance genes were not assigned to individual cultured isolates, and many community members detected by metagenomics were not recovered in culture.

Notably, three isolates exhibited carbapenem resistance, including representatives of *Pseudomonas* spp. and *Acinetobacter junii*. Although rare, the presence of carbapenem-resistant phenotypes within an environmental dental niche underscores the ecological resilience and clinical relevance of these biofilms. Carbapenem resistance in water-associated opportunists has been increasingly linked to hospital plumbing systems and sink-associated biofilms, where oxidative stress and sublethal disinfectant exposure may co-select for intrinsic and acquired resistance mechanisms (85). The detection of such phenotypes in spittoons therefore reinforces their role as potential environmental reservoirs rather than passive collectors of transient oral microbiota.

Importantly, phenotypic resistance was not uniformly distributed across taxa, suggesting that selective pressures within spittoon biofilms favor particular opportunistic lineages capable of both environmental persistence and antimicrobial tolerance. This observation further supports the concept of ecological filtering driving convergence toward stress-adapted, AMR-enriched assemblages.

Although direct transmission events were not assessed, the consistent recovery of viable multidrug-resistant opportunists, combined with the detection of ARGs and plasmid replicons within the metagenomic data set, suggests that dental spittoons merit consideration within environmental surveillance frameworks. Routine monitoring of dental water systems rarely includes spittoon drains despite their direct exposure to patient-derived fluids and source of aerosols. Incorporating these structures into hygiene and disinfection protocols may reduce the persistence of stress-adapted AMR reservoirs within dental healthcare settings.

### Conclusion

This study characterizes dental chair spittoons as structured aquatic biofilm systems shaped by repeated exposure to oral fluids, aerosols, intermittent water flow, and chemical disinfection. Integrated community profiling, genome-resolved metagenomics, and phenotypic susceptibility testing demonstrated that spittoon biofilms are taxonomically differentiated, metabolically redundant, and enriched in disinfectant-tolerant environmental opportunists.

Metagenomic analyses identified a multi-class resistome dominated by β-lactamases and aminoglycoside-modifying enzymes, along with clinically relevant plasmid replicons. Culture-based testing confirmed that a subset of isolates expressed corresponding resistance phenotypes, including multidrug and carbapenem resistance. Together, these findings support the view that dental spittoons can serve as environmental reservoirs of antimicrobial resistance within dental healthcare settings.

Establishing this ecological and resistome baseline provides a framework for incorporating spittoons into dental water system monitoring and biofilm control strategies.

## Data Availability

Negative controls (field blanks, extraction blanks, and no-template controls) were processed alongside samples and inspected for carryover contamination. Taxa or ARGs enriched in controls were excluded. Sequencing data are deposited in the NCBI Sequence Read Archive (SRA) under BioProject accession PRJNA1426735.

## SUPPLEMENTAL MATERIAL

Figure S1 spectrum.00883-26-s0001.tiff Rarefaction curves for 16S amplicon data.

Figure S2 spectrum.00883-26-s0002.tiff Alpha-diversity indices (Chao1 and Pielou evenness).

Figure S3 spectrum.00883-26-s0003.tiff Constrained ordination (dbRDA, Bray-Curtis dissimilarities).

Figure S4 spectrum.00883-26-s0004.tiff Comparison of extraction methods (FastDNA vs ZymoBIOMICS).

Figure S5 spectrum.00883-26-s0005.tiff Differentially abundant genera across departments (DESeq2 analysis).

Figure S6 spectrum.00883-26-s0006.tiff Raup-Crick null-model distribution for spittoon biofilm communities.

Figure S7 spectrum.00883-26-s0007.tiff Relative abundance of major functional categories annotated by DRAM in metagenome-assembled genomes (MAGs) from Orthodontics, Periodontics, and Oral Surgery.

Figure S8 spectrum.00883-26-s0008.tiff Heatmap of DRAM module completeness across departments and dominant taxa.

Supplemental materials spectrum.00883-26-s0009.docx Figures S1 to S9 and Tables S1 to S8.

Table S9 spectrum.00883-26-s0010.xlsx Taxonomic identification and antimicrobial susceptibility profiles of 162 spittoon-derived isolates determined by MALDI-TOF MS and VITEK 2 according to EUCAST criteria.

ASM does not own the copyrights to Supplemental Material that may be linked to, or accessed through, an article. The authors have granted ASM a non-exclusive, world-wide license to publish the Supplemental Material files. Please contact the corresponding author directly for reuse.
