# Supplementary material for: Dental spittoon biofilms as reservoirs of antimicrobial resistance: a longitudinal multi-omics study
Source: Microbiol Spectr. 2026 Sep 21;14(11):e00883-26. doi: 10.1128/spectrum.00883-26 (PMC13729194; doi:10.1128/spectrum.00883-26)
Supplement: Supplementary file 1 [file spectrum.00883-26-s0009.docx]

# Supplementary Figures and Tables

## Supplementary Figures

**Figure S1. Rarefaction curves for 16S amplicon data.** Observed ASV richness as a function of sequencing depth for all samples. The selected rarefaction threshold retained low-biomass samples while capturing the majority of common taxa.

**Figure S2.** Alpha-diversity indices (Chao1 and Pielou evenness). Chao1 (A–B) and Pielou (C–D) diversity indices across departments (Dental prosthesis, Oral surgery, Orthodontics, Periodontics) and sampling months (September 2024–April 2025).

**Figure S3.** Constrained ordination (dbRDA, Bray–Curtis dissimilarities). Distance-based redundancy analysis (dbRDA) constrained by department and sampling time point. Ellipses show 80 % confidence intervals per department. CAP1 = 40.1 % and CAP2 = 21.9 % of explained variation.

**Figure S4.** Comparison of extraction methods (FastDNA vs ZymoBIOMICS). Shannon diversity indices for samples extracted with the FastDNA Spin Kit for Soil and ZymoBIOMICS DNA Miniprep Kit. Wilcoxon p = 0.091; Welch t-test p = 0.154.

**Figure S5. Differentially abundant genera across departments (DESeq2 analysis)**
Heatmap of significant (q < 0.10) log₂ fold-change contrasts between departments at the genus level. Red indicates enrichment, blue depletion relative to the reference group.

## Figure S6. Raup–Crick null-model distribution for spittoon biofilm communities. (A) Histogram showing the distribution of Raup–Crick (Bray–Curtis) values across all pairwise community comparisons. (B) Mean RCbray values per sample grouped by sampling period. Most pairwise comparisons fall within the stochastic range (|RCbray| ≤ 0.95), indicating that community assembly in spittoon biofilms was largely governed by neutral processes such as random dispersal and ecological drift.

**Figure S7.** Relative abundance of major functional categories annotated by DRAM in metagenome-assembled genomes (MAGs) from Orthodontics, Periodontics, and Oral Surgery.
Bars show the proportion of annotated modules per department within core metabolic categories (carbon utilization, membrane transport, nitrogen cycling).

**Figure S8.** Heatmap of DRAM module completeness across departments and dominant taxa.
Modules are grouped by functional class (respiration, carbohydrate metabolism, amino acid synthesis). Color intensity reflects relative completeness per genome bin.

**Figure S9.** Inverse relationship between 16S Shannon diversity and antimicrobial resistance gene (ARG) richness in paired spittoon biofilm samples (Spearman *r* = −0.85, *p* = 0.0035).

**Supplementary Tables**

**Table S1.** Alpha-diversity summary statistics. Kruskal–Wallis tests for alpha-diversity metrics across departments and months.

| **Metric** | **Factor** | **χ²** | **df** | **p-value** |
| --- | --- | --- | --- | --- |
| Shannon | Department | 4.633 | 3 | 0.201 |
|  | Month | 6.07 | 2 | 0.048 * |
| Observed richness | Department | 3.225 | 3 | 0.358 |
|  | Month | 12.36 | 2 | 0.002 ** |
| Chao1 | Department | 3.38 | 3 | 0.337 |
|  | Month | 12.23 | 2 | 0.002 ** |
| Pielou | Department | 7.15 | 3 | 0.067 (ns) |
|  | Month | 17.81 | 2 | 1.3 × 10⁻⁴ *** |

**Table S2.** PERMANOVA of beta-diversity (department × month).

| **Source** | **Df** | **Sum of Squares** | **R²** | **F** | **p-value** |
| --- | --- | --- | --- | --- | --- |
| Model (department x month) | 9 | 5.79 | 0.427 | 1.65 | 0.0002 *** |
| Residual | 20 | 7.78 | 0.573 |  |  |
| Total | 29 | 13.57 | 1.000 |  |  |

**Table S3.** PERMANOVA and dispersion test for the extraction-kit effect.

| **Test** | **Df** | **Sum of Squares** | **R²** | **F** | **p-value** |
| --- | --- | --- | --- | --- | --- |
| PERMANOVA (Kit) | 1 | 0.773 | 0.039 | 1.71 | 0.018 * |
| Residual | 42 | 18.96 | 0.961 |  |  |
| Dispersion (betadisper) | 1 | 0.00295 |  | 0.736 | 0.396 |

**Table S4a.** Sensitivity analysis of the shared core microbiome across prevalence thresholds. Shared genera were defined as taxa detected in all departments at the specified prevalence threshold and ≥0.05% relative abundance. Increasing the prevalence requirement reduced the number of shared genera from 12 at 10% prevalence to three at 50% prevalence, while no genera were shared across all departments at 80% prevalence.

| prevalence_threshold | shared_core_genera_n | shared_core_genera |
| --- | --- | --- |
| 0.1 | 12 | *Methylobacterium–Methylorubrum; Mycobacterium; uncultured; Stenotrophomonas; Elizabethkingia; Brevundimonas; Sphingobium; Bosea; Sphingomonas; Mesorhizobium; A4b; Bifidobacterium* |
| 0.5 | 3 | *Methylobacterium–Methylorubrum; Bosea; Sphingomonas* |
| 0.8 | 0 | *None detected* |

**Table S5.** Indicator-taxon analysis across departments (IndVal.g). Genera were significantly associated with individual departments (p < 0.05).

| **Genus** | **Department** | **IndVal** | **p-value** | **q-value** |
| --- | --- | --- | --- | --- |
| Mycobacterium | Dental Prosthesis | 0.92 | 0.001 | 0.012 |
| Methylobacterium–Methylorubrum | Dental Prosthesis | 0.88 | 0.003 | 0.020 |
| Elizabethkingia | Dental Prosthesis | 0.81 | 0.005 | 0.028 |
| Pseudomonas | Oral Surgery | 0.94 | 0.001 | 0.010 |
| Brevundimonas | Oral Surgery | 0.87 | 0.004 | 0.025 |
| Stenotrophomonas | Oral Surgery | 0.83 | 0.006 | 0.032 |
| Rhodopseudomonas | Orthodontics | 0.90 | 0.002 | 0.018 |
| Bosea | Orthodontics | 0.84 | 0.004 | 0.026 |
| Ensifer | Orthodontics | 0.79 | 0.007 | 0.038 |
| Prevotella | Periodontics | 0.93 | 0.001 | 0.011 |
| Actinomyces | Periodontics | 0.89 | 0.003 | 0.021 |
| Capnocytophaga | Periodontics | 082 | 0.006 | 0.033 |

**Table S6. Summary of metagenomic sequencing and MAG statistics**

| Sample ID | Department | Clean Reads (million) | Data (Gb) | GC (%) | ≥Q20 (%) | ≥Q30 (%) | MAGs recovered | MAG completeness (%) | MAG contamination (%) |
| --- | --- | --- | --- | --- | --- | --- | --- | --- | --- |
| O1 | Orthodontics | 22.5 | 6.42 | 58.7 | 97.3 | 91.8 | 1 | 96.1 | 1.8 |
| O2 | Orthodontics | 24.1 | 6.81 | 59.3 | 97.4 | 92.1 | 1 | 92.7 | 2.5 |
| O3 | Orthodontics | 23.8 | 6.55 | 60.1 | 97.1 | 91.9 | 1 | 88.6 | 2.7 |
| PE1 | Periodontics | 21.6 | 6.41 | 52.4 | 97.8 | 91.5 | 1 | 74.2 | 3.4 |
| PE2 | Periodontics | 22.3 | 6.72 | 51.2 | 97.6 | 91.3 | 1 | 98.1 | 1.1 |
| PE3 | Periodontics | 23.9 | 6.68 | 54.8 | 97.5 | 91.6 | 1 | 93.4 | 1.9 |
| S1 | Oral Surgery | 22.7 | 6.53 | 62.8 | 97.0 | 91.2 | 1 | 89.2 | 2.2 |
| S2 | Oral Surgery | 20.9 | 6.42 | 63.1 | 97.3 | 91.1 | 1 | 84.9 | 3.1 |
| S3 | Oral Surgery | 21.2 | 6.14 | 61.6 | 97.1 | 91.7 | 1 | 72.4 | 3.5 |
| Total / Mean | — | 202.96 M | 60.68 Gb | 56.0 ± 4.7 | 97.3 ± 0.2 | 91.6 ± 0.3 | 9 | 87.7 ± 8.6 | 2.4 ± 0.8 |

**Table S7a. Antimicrobial resistance genes (ARGs) detected by AMRFinderPlus.**

| **Sample ID** | **Department** | **ARG symbol** | **Antibiotic class** | **Gene family / mechanism** | **Coverage (%)** | **Identity (%)** |
| --- | --- | --- | --- | --- | --- | --- |
| **O1** | Orthodontics | *blaCMY* | β-lactam | Class C β-lactamase | 99.0 | 100 |
| **O1** | Orthodontics | *tet(41)* | Tetracycline | Efflux pump | 97.8 | 99.2 |
| **O2** | Orthodontics | *blaSRT* | β-lactam | Class C β-lactamase | 96.2 | 99.0 |
| **O2** | Orthodontics | *aac(6')-Ib* | Aminoglycoside | Acetyltransferase | 95.0 | 99.8 |
| **PE1** | Periodontics | *blaCMY* | β-lactam | Class C β-lactamase | 98.5 | 100 |
| **PE1** | Periodontics | *fosA* | Fosfomycin | Glutathione transferase | 96.0 | 99.0 |
| **PE2** | Periodontics | *blaORN-1* | β-lactam | β-lactamase | 95.6 | 99.5 |
| **PE2** | Periodontics | *aac(3')-IIa* | Aminoglycoside | Acetyltransferase | 97.1 | 98.9 |
| **PE3** | Periodontics | *aph(3')-Ia* | Aminoglycoside | Phosphotransferase | 98.0 | 100 |
| **S1** | Oral Surgery | *ampC* | β-lactam | Class C β-lactamase | 99.3 | 99.8 |
| **S1** | Oral Surgery | *blaOXA* | β-lactam | Class D β-lactamase | 98.7 | 99.1 |
| **S2** | Oral Surgery | *blaPDC-5* | β-lactam | Class C β-lactamase | 96.9 | 98.5 |
| **S3** | Oral Surgery | *blaSRT-2* | β-lactam | Class C β-lactamase | 97.5 | 99.4 |
| **S3** | Oral Surgery | *aac(6')-Ib* | Aminoglycoside | Acetyltransferase | 98.0 | 100 |

**Table S7b.** **Counts per department aggregated at gene symbol level** (AMRFinderPlus/ResFinder).

| **Department** | **Gene** | **Count** |
| --- | --- | --- |
| Oral surgery | fos | 5 |
| Oral surgery | catB | 5 |
| Oral surgery | aac(6') | 3 |
| Oral surgery | blaOXA | 3 |
| Oral surgery | ampC | 3 |
| Oral surgery | blaSRT-2 | 2 |
| Oral surgery | blaPDC-5 | 2 |
| Oral surgery | tet | 2 |
| Oral surgery | aph(3') | 2 |
| Oral surgery | aph(3')-IIb | 2 |
| Oral surgery | aph(3')-IIc | 1 |
| Oral surgery | aac(6')-Iz | 1 |
| Oral surgery | aadS | 1 |
| Oral surgery | arr | 1 |
| Oral surgery | blaGOB | 1 |
| Oral surgery | blaCME | 1 |
| Oral surgery | blaB | 1 |
| Orthodontics | aac(6') | 3 |
| Orthodontics | blaSRT | 3 |
| Orthodontics | fos | 3 |
| Orthodontics | tet(41) | 3 |
| Orthodontics | bla | 2 |
| Orthodontics | blaCMY | 1 |
| Orthodontics | catA | 1 |
| Orthodontics | aph(3')-I | 1 |
| Periodontics | fosA | 4 |
| Periodontics | blaCMY | 3 |
| Periodontics | blaORN-1 | 3 |
| Periodontics | catA | 3 |
| Periodontics | aph(3')-I | 3 |
| Periodontics | oqxB | 3 |
| Periodontics | blaSRT-2 | 1 |
| Periodontics | aac(6') | 1 |

**Table S8.** **Plasmid replicon types detected by PlasmidFinder.**

| **Sample ID** | **Department** | **Plasmid type** | **Identity (%)** | **Query / Template length** | **Contig ID** | **Position (bp)** | **Accession** |
| --- | --- | --- | --- | --- | --- | --- | --- |
| O1 | Orthodontics | IncFII(Yp) | 99.57 | 230 / 230 | k141_1625 | 30952–31181 | CP000670 |
| O1 | Orthodontics | Col440I | 95.15 | 103 / 114 | k141_3677 | 204–306 | CP023920.1 |
| O1 | Orthodontics | IncFIB(K) | 98.75 | 560 / 560 | k141_5180 | 2099–2658 | JN233704 |
| O1 | Orthodontics | RepA | 100.0 | 596 / 596 | k141_2816 | 1791–2386 | CP011611 |
| O2 | Orthodontics | IncFIB(K) | 98.75 | 560 / 560 | k141_28 | 8638–9197 | JN233704 |
| O2 | Orthodontics | IncFII(Yp) | 99.57 | 230 / 230 | k141_104 | 19410–19639 | CP000670 |
| PE1 | Periodontics | RepA | 100.0 | 596 / 596 | k141_2774 | 8934–9529 | CP011611 |
| PE2 | Periodontics | Col440I | 95.15 | 103 / 114 | k141_1169 | 3452–3554 | CP023920.1 |
| PE2 | Periodontics | RepA | 100.0 | 596 / 596 | k141_116 | 9070–9665 | CP011611 |
| PE2 | Periodontics | IncFIB(K) | 98.93 | 560 / 560 | k141_816 | 4265–4824 | JN233704 |
| PE3 | Periodontics | RepA | 100.0 | 596 / 596 | k141_594 | 9070–9665 | CP011611 |
